# Intracellular functions of RNA-binding protein, Musashi1, in stem and cancer cells

**DOI:** 10.1186/s13287-020-01703-w

**Published:** 2020-05-24

**Authors:** Mahboobeh Forouzanfar, Liana Lachinani, Kianoush Dormiani, Mohammad Hossein Nasr-Esfahani, Ali Osmay Gure, Kamran Ghaedi

**Affiliations:** 1 Department of Cell and Molecular Biology and Microbiology, Faculty of Biological Science and Technology, University of Isfahan, Hezar Jerib Ave., Azadi Square, Isfahan, P.O. Code 81746 Iran; 2 Department of Molecular Biotechnology, Cell Science Research Center, Royan Institute for Biotechnology, ACECR, Isfahan, P.O. Code 816513-1378 Iran; 3 Department of Cellular Biotechnology, Cell Science Research Center, Royan Institute for Biotechnology, ACECR, Isfahan, Iran; 4 Department of Molecular Biology and Genetics, Faculty of Science, Bilkent University, Ankara, Turkey

**Keywords:** Cancer stem cells, Cancer progression, Musashi, RNA-binding protein

## Abstract

RNA-binding protein, musashi1 (MSI1), is a main protein in asymmetric cell division of the sensory organ precursor cells, whereas its expression is reported to be upregulated in cancers. This protein is a critical element in proliferation of stem and cancer stem cells, which acts through Wnt and Notch signaling pathways. Moreover, MSI1 modulates malignancy and chemoresistance of lung cancer cells via activating the Akt signaling. Due to the main role of MSI1 in metastasis and cancer development, MSI1 would be an appropriate candidate for cancer therapy. Downregulation of MSI1 inhibits proliferation of cancer stem cells and reduces the growth of solid tumors in several cancers. On the other hand, MSI1 expression is regulated by microRNAs in such a way that several different tumor suppressor miRNAs negatively regulate oncogenic MSI1 and inhibit migration and tumor metastasis. The aim of this review is summarizing the role of MSI1 in stem cell proliferation and cancer promotion.

## Introduction

Musashi, a neural RNA-binding protein, was identified in 1994 with an important role in neural development. This protein is also essential for asymmetrical division and development of sensory organ in *Drosophila melanogaster*. Asymmetric cell division of the sensory organ precursor (SOP) cell in wild type animal generates non-neuronal precursor cell and neuronal precursor cell whereas in *msi1* mutated animal, it generates two non-neuronal precursor cells. As a result of this symmetrical division in *Drosophila*, double hair-shape phonotypes emerge. This gene was termed “Musashi” after Miyamoto Musashi, a famous Japanese samurai who was fighting with two swords [1, 2]. In vertebrates, musashi family have two extremely conserved homolog proteins, MSI1 (Musashi1) and Musashi 2 (MSI2), which were discovered in mice [3, 4]. MSI2 plays important roles in hematopoiesis, and its dysregulated expression is associated with several hematopoietic malignancies [5]. Further studies revealed that *Msi1* is expressed in CNS stem cells and neural stem cells of vertebrates [6, 7]. Human *MSI* gene is located on chromosome 12, which encodes a protein containing two tandems with highly conserved RNA-recognition motifs. Each RNA-binding domain (RBD) is composed of antiparallel β-sheets packed against two α-helices. In vitro selection method, SELEX, demonstrated that MSI1 blocks translation of its target genes by binding to (G/A) Un (AGU) sequence motifs (*n* = 1–3) at 3′UTR of target mRNAs. These motifs are repeated two or three times in 3′UTR regions [8]. The mechanism of MSI1 function is prevention of the initiation of translation of Numb mRNA by competing with eIF4G factor for binding to poly(A) binding protein (PABP) and hindering the construction of 80s ribosome complex [9]. Therefore, MSI1 protein has an important role in activating Notch signaling by targeting *Numb* mRNA expression as an inhibitor of Notch pathway [10].

MSI1 targets several genes, which are involved in the proliferation of stem cells and cell cycle regulation. Cancer stem cells undergo symmetric and asymmetric cell divisions. It is demonstrated that *MSI1* expression increases proliferation of cancer cells in different type of cancers [11, 12]. In the normal state, *MSI1* expression in mammary epithelial cells drives proliferation of mammary stem/progenitor cells by activation of Notch and Wnt pathways. Downregulation of the cyclin-dependent kinase inhibitor p21^Cip1^, Dickkopf-3 (DKK3), and Numb mRNA followed by expression of *MSI1* is responsible for cell proliferation [13]. In this review, we discuss the functional aspects of MSI1 in stem cell biology and cancer development.

### The role of *MSI1* expression in stem cells

Early studies have shown that mouse *Msi1* is highly expressed in CNS progenitor cells and has an important role in brain development. Expression of *Msi1* is also reported in astroglial progenitor cells and mature astrocyte cells [3, 6, 7]. Msi1 is a vital factor for self-renewal maintenance of stem cells. The expression of *Msi1* is required for oligodendrocyte progenitor lineage cell survival and preventing differentiation of oligodendrocyte progenitor cells into mature oligodendrocytes [14]. Indeed, regulation of Msi1 function is necessary for transition cell fate in rat neural stem/progenitor cells (NSPCs). Phosphorylation of regulatory conserved site at serine 337 in MSI1 protein causes differentiation of neural stem/progenitor cells and SH-SY5Y cells by accumulation of p21^WAF1/CIP1^ protein as target mRNA for MSI1. In fact, inhibition of MSI1 protein phosphorylation acts like overexpression of this protein and stop differentiation through regulation of cell cycle inhibitory protein [15].

*MSI1* could be used as a stem cell marker to isolate adult stem cells in intestinal epithelium. Plateroti and colleagues developed transgenic mouse model for targeted expression of *Msi1* in the intestinal epithelium to study the role of *Msi1* in cell cycle and stem cell activity. Expression of stem cell markers *Ccnd1*, *Cdk6*, and *Sox4* were enhanced as a result of targeted *Msi1* overexpression and cell proliferation rate in the intestinal epithelium [16, 17]. A population of active stem cells which called reserve intestinal stem cells (rISCs) are resistant to γ radiation treatment of malignancy. During the regenerative phase after injury induction by γ radiation, the expression level of *Msi1* increases as an inhibitor of p21^Waf1/Cip1^ which promotes proliferation of intestinal stem cells and plays a critical role during regenerative responses [18]. As regards MSI1 function in maintenance of stem cell properties and regenerative phase after damage which mentioned above, the role of this gene in regeneration of lost neural cells in neurodegenerative disease could be interesting for investigation in future.

**Fig. 1:**
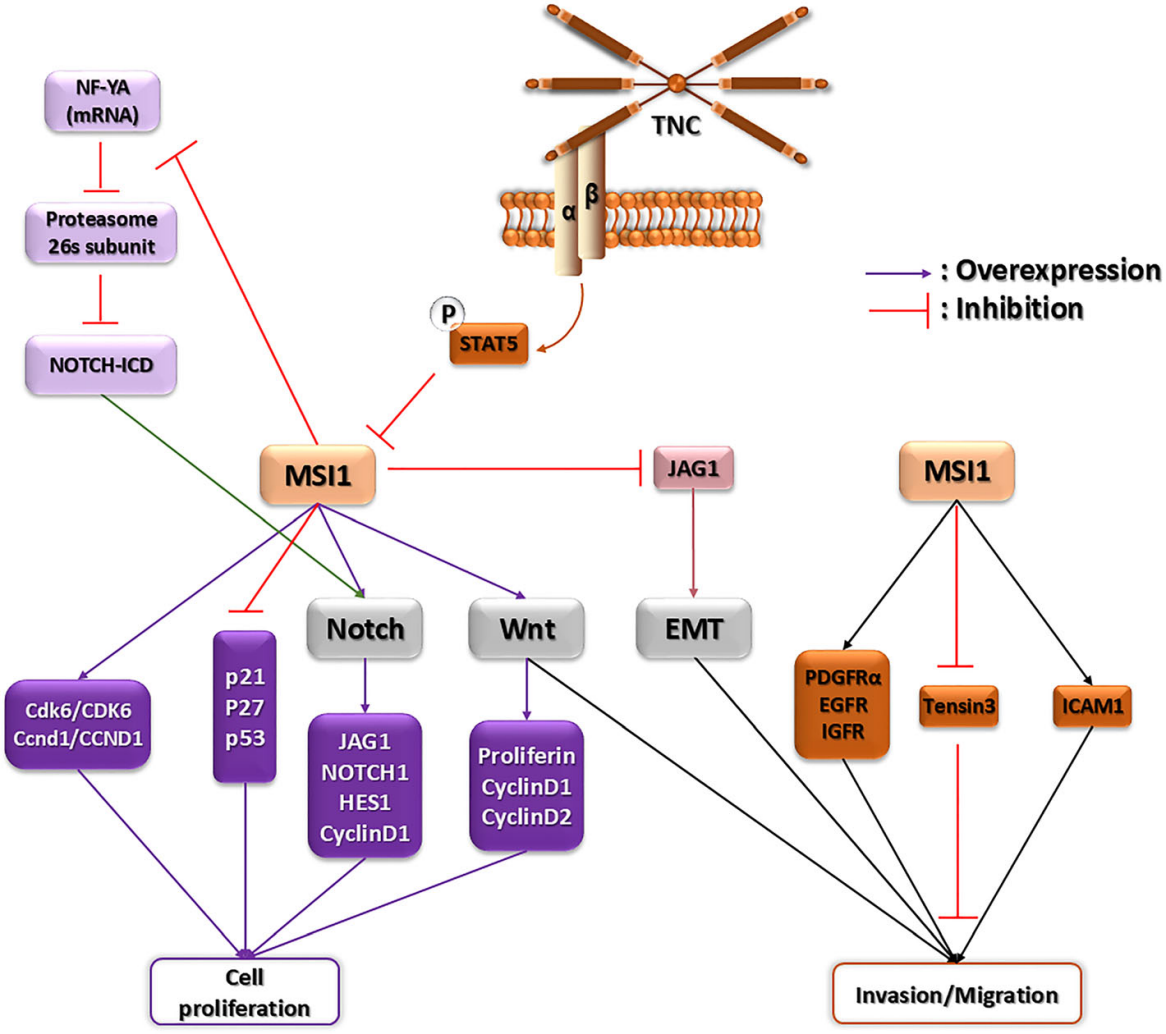
The main signaling pathways for proliferation, invasion, and migration of stem and cancer stem cells in which MSI1 is involved

Furthermore, *Msi1* is highly expressed in spermatogonia and plays a critical role during germ cell development in mouse. Recently, it has been shown that *Msi2* and enhancer of rudimentary homolog (*Drosophila*) (*Erh*) are potential targets of MSI1 through three canonical MSI1 binding sites in their 3′UTR. Subcellular relocation of MSI1 during spermatocyte differentiation prevents activation of *Msi2* and *Erh*. Moreover, MSI1 stabilizes *Msi2* RNA within the cytoplasm of spermatogonia and represses the translation of *Msi2*. Nuclear translocation of MSI1 occurs as a result of interaction between MSI1 and importin-β nuclear import protein, IPO5, which acts as a unique molecular switch for MSI2 release. Therefore, MSI1 is a major regulator of posttranscriptional control during murine germ cell development [19, 20]. Consistent with *Xenopus* findings, MSI1 associates with embryonic poly (A) binding protein family (ePABP) or the canonical somatic cell poly(A) binding protein (PABPC1) and activates translation of target mRNAs in oocyte maturation [21]. Although these studies confirmed that MSI1 is a key component of stem cell development and oocyte maturation, understanding the similar function of MSI1 and its role in human fertility and infertility remains to be obscured. Schematic representation of MSI1 function in stem and cancer stem cells is shown in Fig. 1. In conclusion, a variety of these functions are mentioned in Table 1.

**Table 1:** Diverse roles of Msi1 in different cell

Cell type	Function	Mechanisms	References
Mammary stem/progenitor cells	Cell proliferation	Activation of Notch and Wnt pathways	Imai, 2001; Wang, 2008
Intestinal epithelium cells	Controls of cell cycle and cell proliferation	Stabilizing Ccnd1/CCND1, Cdk6/CDK6 and sox4/SOX4	Cambuli, 2013 & 2015
Oligodendrocyte progenitor cells (OP)	Transition cell fate (Prevention of differentiation)	de-repression of p21WAF1/CIP1	Dobson, 2008; MacNicol, 2015
Germ cell developments	Spermatogenesis and Oocyte maturation	Prevention of MSI2 and Erh RNA-binding activity, Activating translation of ePABP and PABPC1	Sutherland, 2014 & 2015; Cragle, 2019

MSI2, another member of MSI RNA-binding protein family, has a critical role in maintenance of hematopoietic stem cells. Rentas and colleagues revealed that *MSI2* expression attenuates aryl hydrocarbon receptor (AHR) signaling in hematopoietic stem and progenitor cell (HSPC) [22]. On the one hand, the role of MSI2 has been addressed in chemoresistance ability of liver cancer stem cells. Recent study has shown that there is a positive correlation between *MSI2* and *LIN28* expressions in maintenance of stemness properties and chemoresistance of hepatocellular carcinoma (HCC) cancer stem cells [23].

### Association of *MSI1* expression and cancers

RNA sequencing data have shown that the expression level of *MSI1* increased in several types of solid tumors like breast, prostate, lung, and brain tumors while its expression in normal tissues is limited to stem cells [24, 25]. Therefore, it could be concluded that the overexpression of *MSI1* enhances cancer promotion. In cervical cancer, *MSI1* expression increases cancer cell proliferation through regulation of the cell cycle. MSI1 as an RNA-binding protein directly binds to p21, p27, and p53 mRNAs and prohibits translation of checkpoint regulators. As a result, MSI1 promotes G0/G1 to S phase transition [12]. MSI1 controls the proliferation of cancer stem cells by modulating the Notch and Wnt pathways, as well as Akt signaling pathway. It is reported that in spheroid breast cancer cell culture, expression of *MSI1* elevates in CD133^+^ cancer stem cells. By reduction of *MSI1* expression in spheroid culture, proliferation of cancer stem cells decreases through reduction of *Notch1* and cancer stem cell markers such as *Oct4*, *Sox2*, and *c-Myc*. But, the expression level of p21^CIP1^ increases [26]. There is a positive feedback loop between MSI1 and Wnt signaling pathway. Also, there is a functional binding site of T cell factor/lymphoid enhancer factor (TCF/LEF) transcription factor in *MSI1* promoter via in silico analysis. Therefore, the expression of *MSI1* could be regulated by activation of Wnt signaling pathway. Following activation of Wnt signaling pathway through enhancement of activated β-Catenin, expression of target genes like *cyclinD1*, *D2*, and *myc* elevates. On the other hand, by overexpression of *MSI1* in intestinal epithelium stem cells, intracellular domain of the Notch-1 receptor (NICD) and Hes1 upregulate and notch signaling pathway is activated [27]. Downregulation of *MSI1* in glioblastoma cell lines caused prolongation of cell cycle through accumulation of cyclinB1. In addition, enhancement of Numb and PTEN as a result of *MSI* reduction causes deactivation of PI3-Kinase, Akt, and Notch signaling pathways and inhibits cancer cell proliferation in glioblastoma xenograft tumors [28]. Indeed, silencing of *MSI1* inhibits Wnt and Notch pathways and decreases spheroid colony formation in lung cancer [29]. Furthermore, in non-small cell lung carcinoma (NSCLC), MSI1 develops lung cancer through activation of the Akt signaling pathway. By ectopic expression of *MSI1* in NSCLC cell lines, A549 and H522, phosphorylated Akt is increased and cancer cell proliferation is elevated. After inhibition of the Akt pathway through treating cell lines with MK2206 small molecules, proliferation of cancer cells decreases [30]. *MSI1* is also required for tumorigenesis in medulloblastoma and reduction of its expression decreases neurosphere formation, cell proliferation, and tumor growth. Decreasing in *Bcl2 and βIII-tubulin* expression followed by *MSI1* reduction resulted in prevention of differentiation and apoptosis induction [31, 32]. Ribonucleoprotein immunoprecipitation followed by microarray analysis (RIP-chip) indicated that *Small GTP Binding Protein*, *Rac1*, *Connective Tissue Growth Factor (CTGF)*, and *Syntenin* are the major mRNA targets of an MSI1-associated network in U251 glioblastoma cells [32].

Unlike other tumors which progress by metastasis, in glioblastoma the main mechanisms for tumor spread and relapse stage are cell migration and invasion into nearby normal tissues. Invasion depends on intrinsic factors and interaction between cells and extracellular matrix. Gene ontology and pathway analysis have shown that *MSI1* has an important role in multiple processes such as cell adhesion, invasion, and migration. Defect in adhesion pathway following *MSI1* knockdown can give rise to reduction in translation of related proteins including *Platelet-Derived Growth Factor Receptor Alpha* (PDGFRα), *Epidermal Growth Factor Receptor* (*EGFR*), and *Insulin Growth Factor 1 Receptor* (*IGF1R*). Therefore, in glioblastoma, MSI1 has a major role in migration, adhesion, and invasion pathways by interacting with target genes [33]. Recent study about molecular mechanisms of the cell migration in glioblastoma indicated that MSI1 promotes cell migration by inhibition in translation of *Tensin3*, a negative regulator of migration [34], and overexpression of *Intercellular Adhesion Molecule-1* (*ICAM1*) [35]. MSI1 could promote tumor progression through interaction with Ago2 in glioblastoma. Under hypoxic stress, MSI1 recruits cytosolic AGO2 and MSI1/AGO2 complex binds to target mRNAs of cell cycle regulators. This complex destabilizes target mRNAs related to apoptosis. Therefore, MSI1/AGO2 pathway promotes stress-induced tumor growth [36].

Furthermore, silencing of *MSI1* increases DNA damages in cancer cells treated with ionizing radiation through reduction in frequency of non-homologous end-joining (NHEJ) repair. DNA protein kinase catalytic subunit (DNA-PKcs) has a main role in NHEJ, in which its expression is controlled by MSI1. DNA-PKcs, a main factor in NHEJ, is one of the MSI1 target genes which is stabilized by MSI1 and thereby increases the repair of DNA damage and cell survival [37].

Breast cancer is a heterogeneous disease composed of different subtypes of breast cancer cells with different morphological features and clinical behaviors. One of the most abundant subtypes of breast cancer is estrogen and progesterone receptor positive cells (ERα/PR^+^) [24]. ERα/PR^+^ cells are breast epithelial stem cells that preserve self-renewal through asymmetric cell division. It is revealed that loss of the two main regulators of asymmetric cell division, MSI1 and Notch1, in these cells causes creation of ERα/PR^+^ breast cancer [38]. Additionally, MSI1 maintains cancer stem cell phenotype as a consequence of stimulating Notch signaling due to downregulation of 26s proteasome activity. A conserved nuclear transcription factor Y (NF-Y) complex consists of three subunits, NF-YA, NF-YB, and NF-YC. NF-YA regulates expression of 26s proteasome subunit and in both breast cancer and glioma. MSI1 binds mRNA of this factor and decreases its protein level and activity. Eventually, MSI1 downregulates the expression of 26s proteasome subunit [39]. Another mechanism for MSI1 to promote cell growth in breast cancer is stabilizing the target mRNA. In this way, MSI1 stabilizes *Tachykinin1 (TAC1)* mRNA by competing with miR-130a and miR-206. MSI1 binds to noncoding 3′UTR of *TAC1* mRNA within exon 7, therefore causing enhancement of substance P as its major peptide [40].

Contrary to the role of MSI1 in other tumors which increases cancer cell proliferation, in luminal tumors and luminal breast cancer cell lines, MSI1 is responsible for epithelial-luminal transition. On the other hand, MSI1 inhibits epithelial-mesenchymal transition (EMT) by downregulation of *JAGGED1* as a trigger of Notch. Downregulation of *MSI1* by RNAi changes the morphology of luminal breast cancer cells (MCF-7) to basal-like appearance. In addition, epithelial marker expression is decreased while mesenchymal marker expression increases [25]. Therefore, considering the MSI1 role in breast cancer progression, this gene is essential for the preservation of epithelial-luminal transition.

Although the roles of MSI1 in different cancers extensively correlated with proliferation of cancer cells, its role in cancer cell migration and invasion proffers a much broader role. A recent study has shown a positive correlation between MSI1 and epithelial-mesenchymal markers, vimentin, and snail in tissues of cervical cancer [41]. On the other hand, *MSI1* knockdown decreases proliferation of cancer stem cells and induces apoptosis in solid tumors. In lung cancer, MSI1 is a diagnostic marker and is highly expressed in spheroid cultures of tumor cells. Knockdown of *MSI1* in A549 bronchioalveolar carcinoma and NCI-H520 squamous cell carcinoma reduces the spheroid colony proliferation. This phenomenon is accompanied by a decrease of nuclear localization of β-catenin. The enhancement of Numb followed by *MSI1* knockdown inhibits intracellular processing of Notch. On the other way, *MSI1* silencing in oral squamous cell carcinoma cell lines represses cell proliferation and progression through inactivation of the STAT3 signaling pathway [42]. Reduction of *MSI1* expression in breast cancer cell lines decreased the *NOTCH1*, *c-MYC*, *ERBB2*, and *ERK1/2* expressions, which eventually inhibited the survival of tumor cells [26]. Downregulation of *MSI1* increases apoptosis and G2/M arrest in human colon carcinoma HCT 116 cell line and leads to regression of tumor xenografts [43]. In glioblastoma and medulloblastoma cells, the downregulation of *MSI1* induced elongation of mitosis by accumulation of cyclin B1 in addition to the reduction of notch and PI3 kinase-Akt signaling pathways which results in reduction of the cell growth and survival [28].

**Fig. 2:**
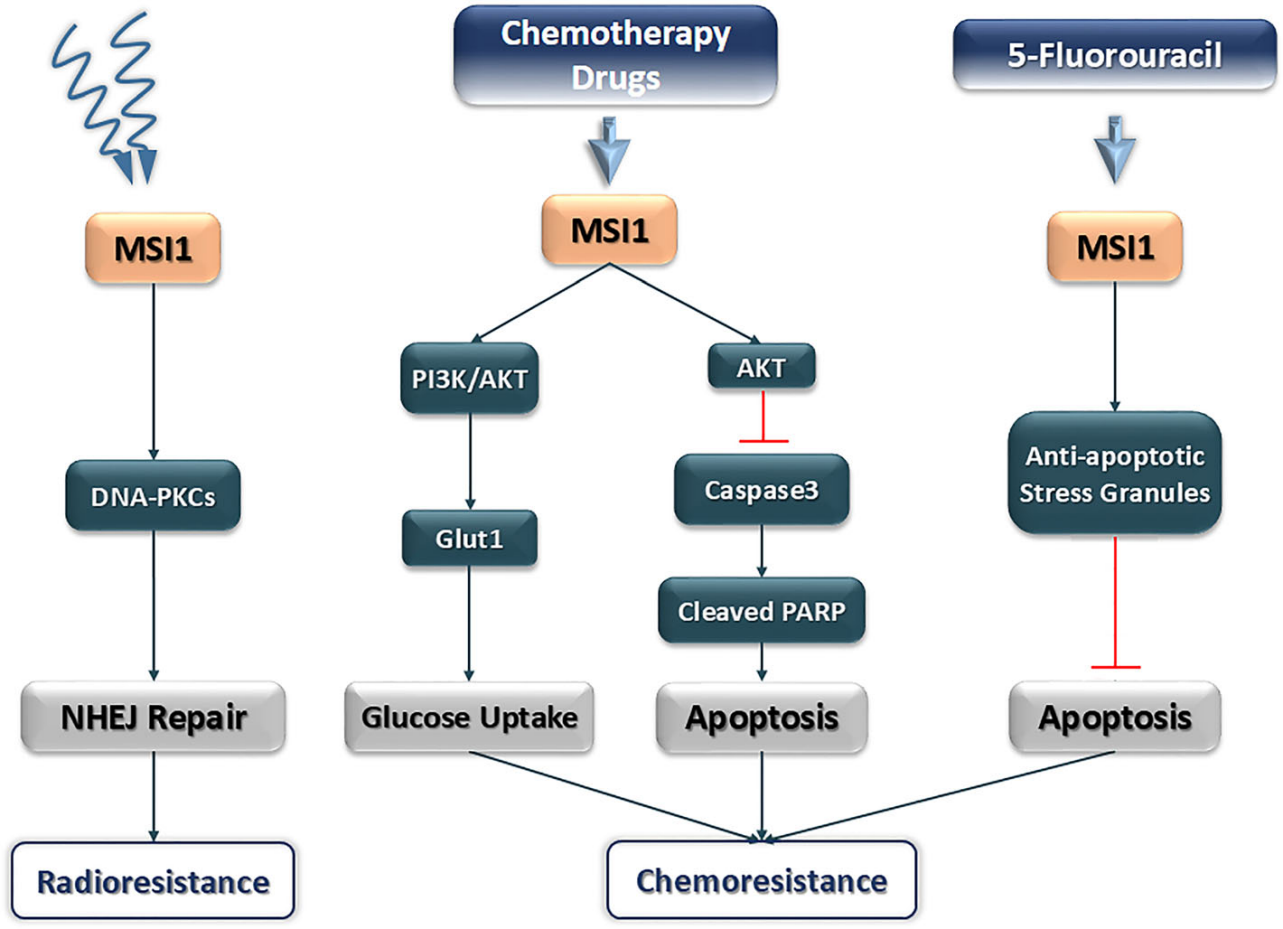
Different signaling pathways which are mediated by MSI1 upon chemotherapy and radiotherapy approaches in a variety of cancers

Overexpression of *MSI1* has been observed in treated cancer cells with chemotherapy drugs and radiotherapy. In drug resistance pathway, MSI1 is effective in protection of cancer cells form apoptosis. Following chemotherapy treatment in glioblastoma, enhancement of *MSI1* expression causes IL-6 secretion through activation of AKT. Secretion of cytokine IL-6 proceeds to a positive regulatory loop between IL-6/AKT and enhanced phosphorylation of AKT on serine 473. Following the MSI1/AKT/IL-6 axis, the apoptosis rate of treated cancer cells diminishes through reduction of caspase3 and cleaved Poly (ADP-ribose) polymerase (PARR) activation [44]. Also, it is revealed that MSI1 induces anti-apoptotic stress granule (SG) formation following 5-fluorouracil treatment in colorectal cancers [45]. Recently, it has been shown that *MSI1* stimulates glucose uptake in non-small cell lung carcinoma (NSCLC) and influences the mitochondrial respiration and aerobic glycolysis affecting the cell oxygen consumption rate (OCR) and extracellular acidification rate (ECAR) in A549 and H522 cells [30]. In hepatocellular carcinoma, expression of *MSI1* elevates. It is already evidenced that overexpression of *MSI1* triggers activation of the Wnt/β-catenin signaling pathway, thereby increasing migration and invasion of hepatocellular carcinoma cells. On the other hand, stemness properties of cancer stem cells in hepatocellular carcinoma are dependent to *MSI1* and *CD44* expression [46] (Figs. 1 and 2). A brief description on diverse functions of MSI1 for tumor progression and malignancy is shown in Table 2.

**Table 2:** The role of MSI1 in promotion of different solid tumors

Type of cancer	Pathways	Function	Target genes	Regulation of ***MSI1*** expression	Reference
Glioblastoma multiform	Notch signaling	Proliferation of glioblastoma; tumorspheroids and neuronal differentiation	–	miR-34a, miR-101, miR-128, miR-137, miR-138	Penalva, 2011
Glioblastoma multiform	Notch and PI3 Kinase/Akt Signaling	Cancer cell growth	Caspase3	–	Okano, 2012
Glioblastoma multiform	Double-strand break repair and nonhomologous end-joining (NHEJ)	Radio-resistance	DNA-protein kinase catalytic subunit (DNA-PKcs)	–	Penalva, 2016
Glioblastoma multiform	AKT signaling and apoptosis	Malignancy and chemoresistance	Caspase3, cleaved PARP	–	Chen, 2016
Colorectal cancer	Wnt and Notch signaling	Cancer progression	Hes1, c-Myc, APC	miR-137	Liang, 2015
Ovarian cancer	–	Tumor progression	p21, p27, and p53	miR-761	Zhang, 2016
Lung cancer	AKT signaling	Malignancy and Chemo resistance	pAKT	miR-181	Xu, 2017
Cervical cancer	Cell cycle checkpoint	Tumor growth and cell proliferation	p21, p27 and p53	–	Zheng, 2014
Breast cancer	Epithelial-mesenchymal transition	Epithelial-luminal cell state	Jagged1	–	Burge, 2014

#### Regulation of *MSI1* in different cancers

The factors that control *MSI1* expression and promote upregulation of the gene in tumors are mostly unknown. Gene expression can be regulated by epigenetic alteration. One of the genes that are highly expressed in breast cancer stem cells is *MSI1*. CpG-rich sites were recognized in the promoter region of *MSI1* gene. Methylation of CpG islands in the *MSI1* promoter has been shown in several primary breast cancer tumors where hypomethylation leads to gene activation [47]. Different factors which stabilize MSI1 could enhance the cancer progress and drug resistance of cancer cells. Tenascin C (TNC), an extracellular matrix protein expressed in stem cell niches of breast cancer, supports pulmonary metastasis. TNC enhances expression of *MSI1* and protects MSI1-dependent Notch signaling by activation of STAT5 [48]. In colorectal cancer cells, the expression of *MSI1* is regulated by Notch 3 signaling. Delta-like 4 (DLL)-4 ligand stimulates Notch1 and increases expression level of *MSI1*. MSI1 as an activator of Notch signaling pathway supports direct activation of Notch1 by DLL4. Activation of Notch1 amplifies transcription of Notch 3 and therefore sustains the circuit [49].

HuR, another important RNA-binding protein is highly expressed in glioblastoma tumors and interacts with 3′UTR of *MSI1* which is rich in U and AU sequences. HuR as an important regulator of *MSI1* stabilizes *MSI1* mRNA and increases its translation. By silencing HuR in U251 and U343 glioblastoma cell lines, the proliferation of cells is reduced and apoptosis is increased, but with ectopic expression of *MSI1*, apoptosis will be rescued [50].

Some small molecules with inhibition of MSI1 function could facilitate to stop cancer cell proliferation and tumor progression. Gossypol as one of these small molecules could interrupt the binding of MSI1 to target mRNA. Gossypol, a natural phenol, binds to RNA-binding domain1 (RBD1) of MSI1 protein to compete with the target mRNA. Consequently, expression levels of *NUMB* and *p21* increases and Wnt and Notch signaling pathways are downregulated. Treatment of colon cancer cell lines with gossypol inhibits cell proliferation and induces apoptosis. Furthermore, gossypol prevents cancer cell line xenograft tumor growth in mouse models [51]. Luteolin is a type of flavonoid which showed a sturdy direct interaction with MSI1. Proliferation of U251 and U343 GBM (glioblastoma) cell lines treated with luteolin was reduced and the treatment repressed migration and invasion of glioblastoma cells. With knockdown of *MSI1* in U251 cells, their sensitivity to luteolin was diminished. So, luteolin is an inhibitor of MSI1 and has an important role in GBM therapy [52].

MicroRNAs are small, noncoding RNAs that control gene expression. MicroRNAs destabilize mRNA by binding to the 3′UTR of target mRNA and hence prevent mRNA translation and protein synthesis [53]. Regulation of some mRNAs at post-translational levels is dependent on polymorphisms at miRNA binding sites in their 3′UTRs. Lately, it was indicated that SNPs’ existence at 3′UTR of target gene in place of binding site for microRNAs has an important role in the development of breast cancer. Interaction of microRNAs with 3′UTR of *ErbB4* mRNA is more frequent when T allele at the rs1836724 position is replaced by C allele [54]. MicroRNAs show oncogenic or tumor suppressor activity in cancerous cells. The expression of some microRNAs are different in several stages of cancer tissues. For instance, miR-9 has an argument expression pattern in different progression levels of breast cancer [55]. Recent data from experimental and computational approaches have shown that cancer pathways are not regulated by single microRNAs but are controlled with some networks of multiple microRNAs [56]. 3′UTR length of target genes for microRNA are approximately 1600 nucleotides whereas 3′UTR length of non-miRNA target genes are smaller and their length is about 1000 nucleotides [57].

**Fig. 3:**
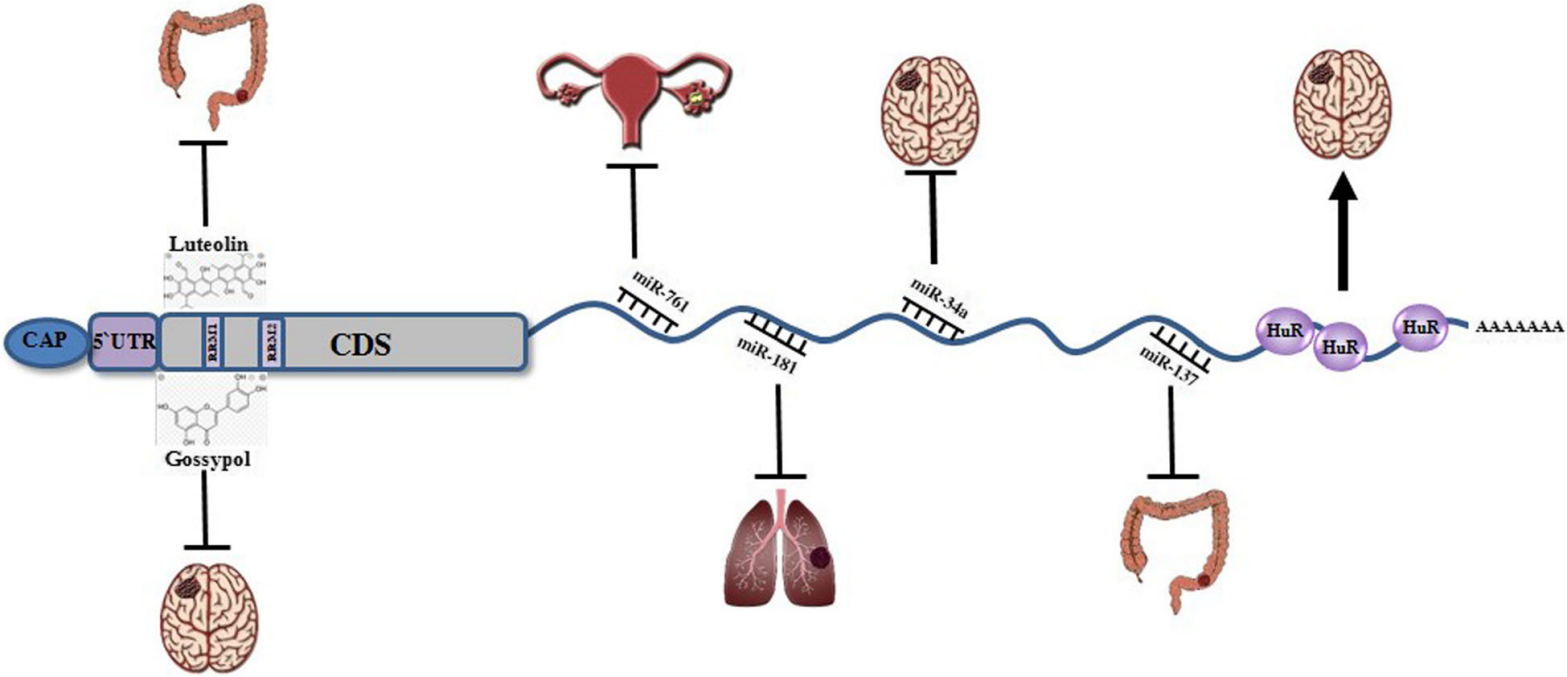
Schematic representation for the functions of MSI1 in various cancer types. Downregulation of Msi1 by binding of tumor suppressor miRNAs could inhibit the growth of tumor cells in different solid tumors. Furthermore, some small molecules like luteolin and gossypol could interact with RNA binding domain1 (RBD1) in CDS of *MSI1* and prevent proliferation of cancer cells and migration. But RNA-binding protein HuR stabilizes *MSI1* and promotes gliblastoma by binding to 3′UTR of *MSI1*

How microRNAs can perform their functional roles in control of diverse cancers progression through regulation of *MSI1* expression will be discussed. *MSI1* RNA comprises long 3′UTR (approximately 1800 nucleotides) which could be controlled by several microRNAs. Tumor suppressor miRNAs (miR-34a, miR-101, miR-128, miR-137, and miR-138) regulate expression of *MSI1* in U251 glioblastoma and Daoy medulloblastoma cells. Transfection of these miRNA mimics into cancer cell lines decrease the cell proliferation. Neuroblastic differentiation assay has shown that after treatment with all-trans retinoic acid and induction of differentiation, expression of *MSI1* was reduced. Conversely, a higher expression of aforementioned tumor suppressor miRNAs was detected after differentiation [58]. It was shown that MSI1 is an activator of Wnt and Notch pathways controlled by miR-133a, miR-138, miR-342, miR-491, and miR-541 in colon carcinoma cell lines [59]. Additionally, in the HCT-116 cell line, miR-137 reduced Wnt and notch signaling pathway as oncogenic pathways and was promoted by MSI1. MSI1 as a tumor suppressor declines the growth of human colon cancer xenograft and inhibits colony formation by cancer cells. The expression of miR-137 in rectal cancer tissue was lower and showed that the loss of miR-137 induced a significant expression of *MSI1* [60]. In ovarian carcinoma, miR-761 inhibits cancer cell proliferation and invasion. Overexpression of miR-761 increases expression of p21, p27, and p53 that are the target of *MSI1* gene [61]. A recent study has shown that *MSI1* was overexpressed in gastric cancer cell lines and gastric cancer tissues. MiR-330 regulates *MSI1* expression in gastric cancer. Consequently, overexpression of miR-330 in HCG-27 cells led to inhibition of cell proliferation and suppression of colony formation. Cell treatment with histone deacetylase inhibitor, trichostatin A (TSA), and DNA methylation inhibitor 5-aza-CdR (AZA) upregulates expression of miR-330. Hence, regulation of miR-330 expression partly was interceded by hypermethylation of the *MSI1* promoter region. As a result, *MSI1* was regulated directly by miR-330 in gastric cancer cells [62]. During studies in non-small cell lung carcinoma, it was demonstrated that *MSI1* is regulated by miR-181a-5p and there is a negative correlation between *MSI1* and miR-181a-5p expressions in NSCLC patients [30]. Nevertheless, an investigation about miRNAs’ role in regulation of *MSI1* regarding inhibition of some cancers like breast cancer is lacking. Some factors which regulate *MSI1* expression in different cancers are represented in Fig. 3.

#### Regulation of *MSI1* in stem cell development

The role of *MSI1* in stem cell development is of interest. Cuadradu and colleagues have confirmed that the thyroid hormone regulates *MSI1* expression in rat brain development. Hypothyroidism in rat causes downregulation of *MSI1* expression in glial and neuron cells. After thyroid hormone injection, in vivo upregulation of *MSI1* was shown in cerebellum. Also, treatment of neuroblastoma N2a cells with T3 hormone shows that *MSI1* amount was increased in the cytoplasm and nucleus. The effect of *MSI1* overexpression on brain development following enhancement of T3 hormone is an augmentation for *Tau* transcript [63]. Furthermore, regulation of *MSI1* through thyroid hormone is shown in adult progenitor cell differentiation during amphibian gastrointestinal remodeling [64].

As mentioned, endogenous expression of *Msi1* is high in mouse neural stem/progenitor cells. Neural stem cell-specific enhancer is positioned on the sixth intron and is involved in neural development. In mouse neural development, expression of *Msi1* is regulated by binding of regulatory transcription factors x (RFx) to regulatory region of *Msi1* which is located in the sixth intron of *Msi1* gene [65, 66].

## Discussion

*MSI1* is a conserved gene during evolution which has a critical role in diverse biological processes such as stem cell proliferation, development, and oncogenesis. MSI1 is essential for proliferation and maintenance of undifferentiated state in neural stem cells. Genome sequencing data in most of cancer tissues compared with normal tissues have shown that *MSI1* was not mutated significantly. But, expression of *MSI1* is increased in at least 40% of breast, prostate, and lung tumors and its profile is similar to oncogenes like *FOS* and *HER2*. Recently, some evidence represented that *MSI1* promotes proliferation and preserves survival of cancer cells in different tumors like glioblastoma, hepatocellular carcinoma, and lung cancer. Enhancement of *MSI1* expression causes tumor spread and relapse. Thus, scientists believe that high *MSI1* is an indicator of poor survival and poor prognosis [26, 33]. *MSI1* is a main therapeutic target because it impacts a broad group of cancers [25]. Chemotherapy is vital for improvement of clinical outcome in cancer patients; however, different studies have shown that MSI1 contributes to chemoresistance in cancer therapy [45]. It was demonstrated that the expression level of *MSI1* was amplified followed by cis-diammine dichloroplatinum (CDDP) therapy in A549 and H522 cell lines. On the other hand, MSI1 controls carcinoma malignancy and chemoresistance through modifying the activity of the Akt signaling pathway in non-small cell lung carcinoma. Inhibition of *MSI1* increased the efficiency of radiotherapy and the overexpression of *MSI1* induced chemoresistance in glioblastoma [37, 44]. Considering the important role of *MSI1* in DNA repair, resistance to chemotherapy, and spread of cancer through normal tissues, *MSI1* targeting using different approaches may have the potential application as a reliable strategy for improvement of cancer treatment.

## Data Availability

Not applicable.

## Abbreviations

AHR: Aryl hydrocarbon receptor
AZA: DNA methylation inhibitor 5-aza-CdR
CDDP: Cis-diammine dichloroplatinum
CTGF: Connective tissue growth factor
DKK3: Cyclin-dependent kinase inhibitor p21^Cip1^, Dickkopf-3
DLL-4: Delta-like 4
DNA-PKcs: DNA protein kinase catalytic subunit
ECAR: Extracellular acidification rate
EGFR: Epidermal growth factor receptor
EMT: Epithelial-mesenchymal transition
ePABP: Embryonic the poly (A) binding protein family
Erh: Enhancer of rudimentary homolog
HCC: Hepatocellular carcinoma
HSPC: Hematopoietic stem and progenitor cell
ICAM1: Intercellular adhesion molecule-1
IGF1R: Insulin growth factor1 receptor
Msi1: Musashi1
NICD: Intracellular domain of the Notch-1 receptor
NF-YA: Nuclear transcription factor Y subunit alpha
NHEJ: Nonhomologous end-joining
NSCLC: Non-small cell lung carcinoma
NSPCs: Neural stem/progenitor cells
OCR: Oxygen consumption rate
PABP: Poly (A) binding protein
PABPC1: Canonical somatic cell poly(A) binding protein
PARP: Poly (ADP-ribose) polymerase
PDGFRα: Platelet-derived growth factor receptor alpha
RBD: RNA-binding domain
RFX transcription factors: Regulatory factor X transcription factors
RIP-chip: Ribonucleoprotein immunoprecipitation followed by microarray analysis
rISCs: Reserve intestinal stem cells
SGs: Stress granules
SOP: Sensory organ precursor
TAC1: Tachykinin1
TCF/LEF: T cell factor/lymphoid enhancer factor
TNC: Tenascin C
TSA: Trichostatin A
